# Neuroimaging Markers Associated With Recurrent Stroke in Intracerebral Hemorrhage and Atrial Fibrillation: Secondary Analysis of PRESTIGE-AF

**DOI:** 10.1212/WNL.0000000000214386

**Published:** 2025-11-06

**Authors:** Simon Fandler-Höfler, Stefan Ropele, Thomas Gattringer, Melanie Haidegger, Daniela Pinter, Markus Kneihsl, Eleni Korompoki, Joan Montaner, Valeria Caso, Peter Arthur Ringleb, Igor Sibon, Omid Halse, Kirsten Harvey, Cornelia Fießler, C.D.A. Wolfe, Peter U. Heuschmann, Roland Veltkamp, Christian Enzinger, for the PRESTIGE-AF Consortium

**Affiliations:** 1 Department of Neurology, Medical University of Graz, Austria;; 2 Division of Neuroradiology, Vascular and Interventional Radiology, Department of Radiology, Medical University of Graz, Austria;; 3 Department of Brain Sciences, Imperial College London, United Kingdom;; 4 Department of Clinical Therapeutics, National and Kapodistrian University of Athens, Alexandra Hospital, Greece;; 5 Institute de Biomedicine of Seville, IBiS/Virgen Macarena University Hospital/CSIC/University of Seville, Spain;; 6 Department of Neurology/Stroke Unit, Saronno Hospital-ASST Valle Olona, Italy;; 7 Stroke Unit and Division of Cardiovascular Medicine, University of Perugia, Italy;; 8 Department of Neurology, Heidelberg University Hospital, Germany;; 9 Department of Neurology, CHU Bordeaux, Bordeaux University, France;; 10 Clinical Trial Center, University Hospital Würzburg, Germany;; 11 School of Life Course and Population Sciences, King's College London, United Kingdom;; 12 Institute for Clinical Epidemiology and Biometry (ICE-B), University of Würzburg, Germany; and; 13 Department of Neurology, Alfried-Krupp Krankenhaus, Essen, Germany.

## Abstract

**Background and Objectives:**

Anticoagulation in patients with intracerebral hemorrhage (ICH) and atrial fibrillation reduces the risk of ischemic stroke (IS), but also potentially causes an excess risk of recurrent ICH. In this context, we assessed the role of neuroimaging in identifying patients with particular susceptibility to IS or ICH, potentially allowing for individualized risk stratification.

**Methods:**

In PRESTIGE-AF, a prospective, randomized clinical trial conducted across 75 hospitals in 6 European countries, participants with spontaneous ICH and atrial fibrillation were randomized to treatment with direct oral anticoagulants or no anticoagulation. Within a prespecified imaging subanalysis, we centrally assessed brain CT and MRI scans at baseline using established scales and definitions. We performed Cox regression analyses to investigate associations of neuroimaging findings with recurrent ICH and IS.

**Results:**

PRESTIGE-AF included 319 patients, and 313 had neuroimaging of sufficient quality (median age 79 years, 35.5% female). MRI was available in 170 patients (54.3%). During a median follow-up of 1.4 years, 13 patients had recurrent ICH and 22 patients had IS. ICH recurrence was not associated with lobar vs nonlobar hematoma location or the overall category of probable cerebral amyloid angiopathy (*p* > 0.2). However, there was an increased risk of recurrent ICH in patients with cortical superficial siderosis (hazard ratio [HR] 7.7, 95% CI 1.4–42.2) and chronic intracerebral macrohemorrhages on MRI (HR 9.1, 95% CI 1.8–46.8). Patients with nonlobar ICH were at increased risk of IS (HR 9.1, 95% CI 1.2–67.7).

**Discussion:**

Neuroimaging contributes to the identification of patients with ICH and atrial fibrillation at particularly high risk of recurrent ICH or IS, emphasizing its role in clinical decision making. MRI allows for the assessment of relevant neuroimaging markers that can aid the risk assessment in affected patients. Major study limitations include the modest number of outcome events, with confirmation of our findings needed in larger collaborations.

**Trial Registration Information:**

ClinicalTrials.gov NCT03996772; first submitted June 21, 2019; first patient enrolled: May 31, 2019; available at clinicaltrials.gov/study/NCT03996772.

## Introduction

Up to a fourth of patients with intracerebral hemorrhage (ICH) exhibit the comorbidity of atrial fibrillation (AF).^1^ Survivors of ICH have a high risk of further cerebrovascular events, in particular those with AF.^2^ While it is known that treatment with direct oral anticoagulants (DOACs) reduces the risk of ischemic stroke (IS) in patients with AF, it may also increase the risk of recurrent ICH, as shown in a recent meta-analysis of pilot randomized controlled trials (RCTs).^3^ The recently published randomized controlled PRESTIGE-AF trial in patients with ICH and AF showed a decreased risk of IS, but an excess risk of recurrent ICH in patients randomized to DOACs.^4^

Most ICHs are caused by cerebral small vessel diseases (SVDs) such as arteriolosclerosis and cerebral amyloid angiopathy (CAA), and the risk of recurrent ICH varies strongly based on the underlying etiology.^5^ Patients with CAA, and particularly those with cortical superficial siderosis (cSS), have a very high risk of recurrent ICH,^6-8^ especially in the first months after the index ICH.^9^ The ongoing ENRICH-AF RCT stopped including patients with lobar ICH (approximately half of which is caused by CAA) because of excess risk of recurrent ICH.^10^ This indicates that risk-benefit analysis of anticoagulation may vary strongly among individual patients depending on their risk profiles, which can be refined using neuroimaging.

Based on these premises, we performed a thorough analysis of neuroimaging parameters of patients included in the PRESTIGE-AF trial, assessing whether specific findings are associated with an excess risk of recurrent ICH or IS.

## Methods

PRESTIGE-AF was a multicenter, open-label, randomized, phase 3 trial performed at 75 centers across 6 countries in Europe, enrolling patients between May 31, 2019, and November 30, 2023. PRESTIGE-AF enrolled adult patients with spontaneous ICH and AF and randomized them 1:1 to receive a DOAC or no anticoagulation. Treatment allocation was known to patients and clinicians, and outcome assessment was performed by an event adjudication committee blinded to treatment allocation. Details of the trial design and inclusion and exclusion criteria were published previously.^4,11^

Neuroimaging confirmation of the index ICH was mandatory. Baseline brain MRI at study inclusion was not required, but strongly recommended. Neuroimaging was centrally assessed at the imaging core laboratory (Medical University of Graz) and performed blinded to clinical variables including outcome events by a single expert rater (S.F.-H.). In unclear cases, a second expert rater (C.E.) was involved to find a consensus rating.

We investigated ICH location according to the Cerebral Hemorrhage Anatomical RaTing inStrument^12^ and assessed hematoma size, concomitant subarachnoid and intraventricular hemorrhage, and evidence of previous ICH (chronic, usually ovoid lesions >10 mm with susceptibility artifacts consistent with hemosiderin deposition) or ischemic infarcts (lacunes were considered separately). SVD-related changes were assessed based on the STRIVE-2 criteria^13^ and included periventricular and deep white matter hyperintensities on MRI as well as white matter hypoattenuation/hypodensities (leukoaraiosis) on CT according to the Fazekas scale,^14,15^ presence of lacunes, severity and distribution of cerebral microbleeds (rated according to the Microbleed Anatomical Rating Scale),^16^ presence and severity of cSS, and enlarged perivascular spaces in the centrum semiovale and basal ganglia according to a validated 4-point scale.^17^ ICH size and concomitant subarachnoid and intraventricular hemorrhage were assessed on the first available neuroimaging (CT or MRI). Further neuroimaging parameters were assessed on MRI if available. If MRI was not available, ICH location, evidence of old infarcts, leukoaraiosis, and lacunes were rated on CT.

The presence of probable CAA was defined based on Boston criteria version 2.0^18^ if MRI was available, otherwise based on the simplified Edinburgh CT criteria (presence of both finger-like projections and subarachnoid hemorrhage indicating “high probability” of CAA).^19^ We further used a modified MRI-based version of the CLAS-ICH classification of ICH etiology, differentiating between CAA, arteriolosclerosis, mixed location SVD, and cryptogenic CAA, as previously described.^20^

The primary outcomes for this planned secondary analysis of the PRESTIGE-AF trial were recurrent ICH and IS during the follow-up period, which was at least 6 months (up to 3 years). We classified the location of recurrent ICH based on clinical and neuroimaging hospital records and defined the most likely underlying etiology of IS also based on these records. Subcortical infarcts <20 mm in the territory of 1 perforating artery with consistent clinical symptoms were defined to be SVD-related while other infarct patterns were deemed to be cardioembolism-related (as all patients in this study had AF), unless competing causes were detected.

### Statistical Analysis

We performed statistical analyses using IBM SPSS Statistics for Windows, version 29 (IBM Corp., Armonk, NY). Before using Cox regression to estimate hazard ratios (HRs) for factors potentially associated with the risk of (first) ICH recurrence and IS, the proportional hazards assumption was verified through visual inspection of log-log survival plots and Schoenfeld residuals. *p* Values of less than 0.05 were considered statistically significant. Owing to the low number of outcome events, we were not able to correct for potential co-factors or investigate treatment subgroups.

### Standard Protocol Approvals, Registrations, and Patient Consents

Research ethics committees and competent authorities in each participating country approved this study. Written informed consent was obtained from patients or their legal representatives. PRESTIGE-AF was registered at ClinicalTrials.gov (NCT03996772).

### Data Availability

Data will be available to researchers on request to the principal investigator of PRESTIGE-AF (r.veltkamp@imperial.ac.uk), subject to approval by an independent review committee identified for this purpose.

## Results

Of the entire cohort of 319 patients included in the PRESTIGE-AF study, neuroimaging of sufficient quality for central analysis was available in 313 patients. Their median age at index ICH was 79 years (interquartile range [IQR] 73–83 years), and 35.5% of patients were female. The most prevalent vascular risk factors were arterial hypertension (95.8%) and diabetes mellitus (24.9%).

**Figure 1:**
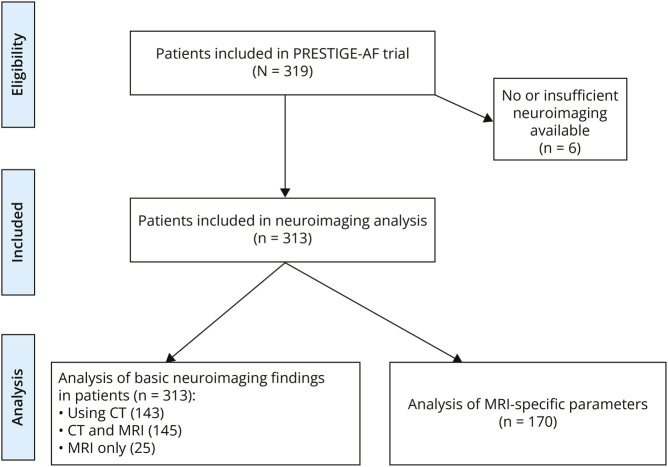
Study Flowchart

A total of 170 patients (54.3%) had a brain MRI available for centralized rating; in the other 143 patients (45.7%), neuroimaging analysis was performed on CT only. A study flowchart is illustrated in Figure 1. The analyzed CT scans had been performed at ICH onset and MRI was mostly performed after the acute stage (median 47 days from ICH diagnosis to MRI, IQR 29–89 days) but usually before study enrollment and randomization (97%). Only 3% of patients had MRI performed after randomization (range 2–42 days).

A total of 94 patients (30.0%) had a lobar ICH, and 219 (70.0%) had a nonlobar ICH (56.5% supratentorial deep, 8.6% cerebellar, 2.2% brainstem). The median follow-up period was 1.4 years (IQR 0.7–2.3 years), and the total follow-up time was 473 patient-years.

### Recurrent ICH

Thirteen patients had a recurrent ICH during the follow-up period. Eleven of those were randomized to the DOAC group and under DOAC treatment at the time of recurrent ICH; 1 patient was randomized to the no-anticoagulation group and was not on DOAC treatment; 1 patient was randomized to the no-anticoagulation group, had a previous IS in the follow-up period, and was consequently treated with DOAC at the time of the recurrent event. Two recurrent ICHs were lobar and 9 were nonlobar, and 2 patients had simultaneous lobar and nonlobar ICH.

Age, sex, or clinical risk factors were not associated with recurrent ICH (Table 1). Presence of CAA (probable CAA based on the Boston criteria 2.0 or high-probability CAA according to the simplified Edinburgh criteria) was not associated with recurrent ICH. Presence of lacunes was associated with recurrent ICH (HR 3.19, 95% CI 1.07–9.50).

**Table 1:** Clinical and Neuroimaging Findings and Their Associations With Recurrent ICH

	Recurrent ICH (n = 13)	No recurrent ICH (n = 300)	*p* Value
Demographic and clinical data			
Age, y, median (IQR)	80 (74–82)	79 (73–83)	0.94
Female sex	6 (46.2)	106 (35.3)	0.45
Arterial hypertension	11 (84.6)	289 (96.3)	0.11
Diabetes mellitus	5 (38.5)	73 (24.3)	0.15
History of ischemic stroke	2 (15.4)	59 (19.7)	0.54
Treatment group: direct oral anticoagulants	11 (84.6)	145 (48.3)	0.02
Neuroimaging findings (CT and/or MRI)			
ICH volume, mL, median (IQR)	1.3 (0.4–11.2)	3.8 (1.4–9.0)	0.52
Lobar ICH location	3 (23.1)	91 (30.3)	0.57
Subarachnoid hemorrhage	0	32 (10.7)	0.45
Presence of lacunes	7 (53.8)	80 (26.7)	0.04
Deep leukoaraiosis (Fazekas scale)	2 (1–3)	1 (1–2)	0.20
Periventricular leukoaraiosis (Fazekas scale)	2 (1–3)	1 (1–2)	0.61
Presence of old cortical or cerebellar infarcts	0	45 (15.0)	0.32
Probable cerebral amyloid angiopathy	2 (15.4)	44 (14.7)	0.98
Time to first CT, d, median (IQR)	0 (0–0)	0 (0–0)	0.70
MRI-specific findings (n = 170)	n = 7	n = 163	
Presence of any cerebral microbleeds^a^	5 (71.4)	121 (78.6)	0.40
Presence of ≥5 cerebral microbleeds^a^	3 (42.9)	54 (35.1)	0.55
Presence of any lobar cerebral microbleeds^a^	4 (57.1)	97 (63.0)	0.84
Presence of any deep cerebral microbleeds^a^	2 (28.6)	67 (43.5)	0.59
Presence of cortical superficial siderosis^a^	2 (28.6)	9 (5.8)	0.02
Presence of old hemorrhages^a^	3 (42.9)	18 (11.7)	0.008
White matter hyperintensities multispot pattern	1 (14.3)	20 (12.3)	0.92
Severe enlarged perivascular spaces, centrum semiovale^b^	5 (83.3)	96 (70.1)	0.73
Severe enlarged perivascular spaces, basal ganglia^b^	3 (50.0)	51 (38.1)	0.58
Diffusion-weighted imaging–positive lesions^c^	0	18 (11.5)	0.56
Time to first MRI, d, median (IQR)	7 (2–36)	6 (2–21)	0.53
Treatment group: direct oral anticoagulants	6 (85.7)	79 (48.5)	0.09
MRI-based classification of ICH etiology^d^			0.26
Cerebral amyloid angiopathy	2 (28.6)	33 (20.4)	
Mixed location cerebral small vessel disease	4 (57.1)	74 (45.7)	
Arteriolosclerosis (deep perforator arteriopathy)	1 (14.3)	40 (24.7)	
Cryptogenic and other	0	15 (9.3)	

Abbreviations: ICH = intracerebral hemorrhage; IQR = interquartile range.

Data are presented as n (%), unless otherwise specified.

a Missing in 9 patients (n = 161).

b Missing in 27 patients (n = 143).

c Missing in 7 patients (n = 163).

d Missing in 1 patient (n = 169).

In the MRI analysis (n = 170, 7 patients with recurrent ICH), patients with cSS had a higher risk of recurrent ICH (HR 7.7, 95% CI 1.4–42.2). Presence of an old macrohemorrhage was also associated with a higher risk (HR 9.1, 95% CI 1.8–46.8). No clear association was found with the presence of probable CAA based on the Boston criteria 2.0 alone (HR 1.44, 95% CI 0.28–7.5, *p* = 0.66). No association was found for presence, severity, and distribution of cerebral microbleeds or enlarged perivascular spaces (Table 1).

**Figure 2:**
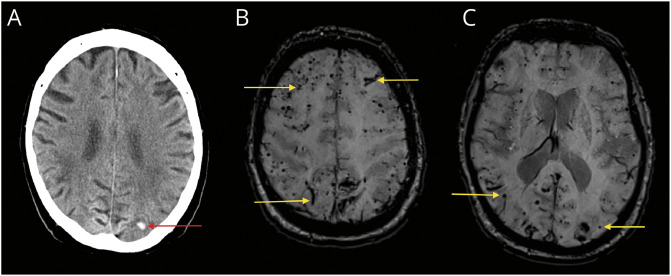
Exemplary Patient With Recurrent ICH Patient in their seventies with a small left occipital intracerebral hemorrhage (A). On susceptibility-weighted imaging, cortical superficial siderosis and numerous lobar cerebral microbleeds were visible (B, C). This patient had 2 recurrent ICH during the follow-up period. ICH = intracerebral hemorrhage.

Figure 2 shows neuroimaging from the only patient in the study with 2 recurrent lobar ICHs, who had severe CAA with disseminated cSS.

### Ischemic Stroke

Twenty-two patients had an IS during follow-up (14 with available MRI). Twenty were in the no-anticoagulation group, although 2 of those were under DOAC treatment at the time of IS (1 due to a previous peripheral arterial ischemic event during the follow-up period, 1 for unknown reasons). Of the 2 patients in the DOAC group, 1 had discontinued treatment at time of the IS because of a previous recurrent ICH. Of the 22 with IS, 14 (63.6%) were considered cardioembolic and 8 (36.4%) were caused by SVD.

There was no association of age, sex, and clinical risk factors with IS (Table 2). Patients with a nonlobar ICH had a higher risk of IS (HR 9.1, 95% CI 1.23–67.7). Eight of 21 IS events in patients with a nonlobar ICH were associated with SVD, and 13 of 21 were due to cardioembolism; no IS in patients with a lobar ICH was associated with SVD (1 caused by cardioembolism). Other than ICH location, no neuroimaging markers were associated with risk of IS.

**Table 2:** Clinical and Neuroimaging Findings and Their Associations With Ischemic Stroke After Index ICH

	Ischemic stroke (n = 22)	No ischemic stroke (n = 291)	*p* Value
Demographic and clinical data			
Age, y, median (IQR)	81 (74–85)	78 (73–83)	0.99
Female sex	10 (45.5)	102 (35.1)	0.38
Arterial hypertension	21 (95.5)	279 (95.9)	0.61
Diabetes mellitus	7 (31.8)	71 (24.4)	0.29
History of ischemic stroke	4 (18.2)	57 (19.6)	0.72
Treatment group: direct oral anticoagulants	2 (9.1)	154 (52.9)	0.002
Neuroimaging findings (CT and/or MRI)			
ICH volume, mL, median (IQR)	3.2 (1.6–8.4)	3.8 (1.3–9.1)	0.84
Lobar ICH location	1 (4.5)	93 (32.0)	0.03
Subarachnoid hemorrhage	0	32 (11.0)	0.30
Presence of lacunes	6 (27.3)	81 (27.8)	0.96
Deep leukoaraiosis (Fazekas scale)	1 (1–2)	1 (1–2)	0.40
Periventricular leukoaraiosis (Fazekas scale)	1 (1–2)	1 (1–2)	0.81
Presence of old cortical or cerebellar infarcts	3 (13.6)	42 (14.4)	0.82
Probable cerebral amyloid angiopathy	2 (9.1)	44 (15.1)	0.44
Time to first CT, d, median (IQR)	0 (0–0)	0 (0–0)	0.86
MRI-specific findings (n = 170)	n = 14	n = 156	
Presence of any cerebral microbleeds^a^	12 (92.3)	114 (77.0)	0.27
Presence of ≥5 cerebral microbleeds^a^	6 (46.2)	51 (34.5)	0.38
Presence of any lobar cerebral microbleeds^a^	8 (61.5)	93 (62.8)	0.99
Presence of any deep cerebral microbleeds^a^	6 (46.2)	63 (42.6)	0.72
Presence of cortical superficial siderosis^a^	0	11 (7.4)	0.54
Presence of old hemorrhages^a^	3 (23.1)	18 (12.2)	0.22
White matter hyperintensities multispot pattern	2 (14.3)	19 (12.2)	0.73
Severe enlarged perivascular spaces, centrum semiovale^b^	2 (22.2)	52 (39.7)	0.29
Severe enlarged perivascular spaces, basal ganglia^b^	7 (77.8)	94 (70.1)	0.80
Diffusion-weighted imaging–positive lesions^c^	1 (7.7)	17 (11.3)	0.63
Time to first MRI, d, median (IQR)	4 (0–20)	7 (3–35)	0.08
Treatment group: direct oral anticoagulants	1 (7.1)	84 (53.8)	0.02
MRI-based classification of ICH etiology^d^			0.55
Cerebral amyloid angiopathy	1 (7.1)	34 (21.9)	
Mixed location cerebral small vessel disease	7 (50.0)	71 (45.8)	
Arteriolosclerosis (deep perforator arteriopathy)	6 (42.9)	35 (22.6)	
Cryptogenic and other	0	15 (9.7)	

Abbreviations: ICH = intracerebral hemorrhage; IQR = interquartile range.

Data are presented as n (%), unless otherwise specified.

a Missing in 9 patients (n = 161).

b Missing in 27 patients (n = 143).

c Missing in 7 patients (n = 163).

d Missing in 1 patient (n = 169).

## Discussion

In this preplanned subanalysis of the PRESTIGE-AF trial, we identified several potential neuroimaging markers associated with increased risk of recurrent ICH and IS. These may be helpful in clinical situations to determine subgroups of patients who might have particular benefit or harm from initiation of DOACs after ICH with concomitant AF.

Patients with cSS had a substantially elevated risk of recurrent ICH, although the quantification of the effect size was imprecise. cSS is the imaging marker of CAA associated with a vastly increased risk of ICH, with the recurrence risk of ICH in patients with cSS exceeding 20% in the first year after index ICH in observational studies.^7,8^ Accordingly, the risk-benefit ratio of initiation of DOACs in patients with cSS may point toward different therapeutic options. Left atrial appendage occlusion has been suggested as a good option for these patients,^21^ but this requires confirmation in ongoing trials (A3ICH NCT03243175; STROKECLOSE NCT02830152; CLEARANCE NCT04298723). Analogue considerations may be applied in patients with ICH and detection of chronic ICHs by MRI, which seems to constitute a marker of particularly high risk of ICH recurrence, with the largest estimated effect size detected in this study. Both these findings can only be reliably detected on MRI, reinforcing the utility of MRI for the understanding of underlying ICH etiologies and long-term risk assessment in affected patients.^5^

Somewhat surprisingly, we did not observe an association of CAA or lobar ICH location with increased risk of recurrent ICH. While this is in line with the pilot RCTs SoSTART^22^ and APACHE-AF,^23^ the similar ENRICH-AF study randomizing patients with AF and ICH to the DOAC edoxaban or no anticoagulation had to stop enrolling patients with lobar ICH and convexity subarachnoid hemorrhage (which are most frequently caused by CAA) because of an excessive risk of ICH recurrence in these patient groups in an interim analysis.^10^ Possible explanations of these somewhat contrary findings include differences in the patient composition (PRESTIGE-AF did not include patients with convexity subarachnoid hemorrhage, which is commonly caused by CAA in elderly populations with AF), the modest number of patients with lobar ICH included in PRESTIGE-AF (and, therefore, even lower numbers of patients with CAA, which is estimated to cause approximately half of lobar ICH), and the weaker effect sizes of CAA on ICH recurrence compared with cSS, which indicates high-risk CAA.

There is an ongoing debate whether anticoagulation causes ICH or only exacerbates the severity of hemorrhages that would have occurred regardless. Previous studies have indicated that severe SVD is associated with higher risks of anticoagulation-associated ICH,^24^ and a recent retrospective study found a lower rate of CAA among patients with anticoagulant-associated ICH compared with anticoagulation-naïve patients. These results indicate that anticoagulation might mediate the risk of ICH differently based on the underlying type and severity of SVD,^25^ but more definitive evidence is missing. What is clear, however, is that anticoagulation-associated ICH has larger hematoma volumes, higher rates of hematoma expansion, and increased mortality compared with ICH in patients not receiving anticoagulation.^26-29^

Patients included in this study were relatively old and had severe cerebral SVD, as suggested by neuroimaging (e.g., presence of cerebral microbleeds in 78%). “Mixed location” patterns of SVD (e.g., hemorrhagic changes in both deep and lobar locations) were found in approximately half of the patients, indicating severe underlying arteriolosclerosis in many and coexisting CAA and arteriolosclerosis in others.^5^ The presence of lacunes was also associated with recurrent ICH and might be considered as a marker of severe underlying SVD.

Patients with nonlobar ICH had increased risks of IS and could constitute a subgroup where the risk-benefit balance of DOAC (re)initiation might tend toward using DOACs. In these patients in whom the ICH is most frequently caused by arteriolosclerosis, 2 possible high-risk pathomechanisms for ISs coexist: cardioembolism (due to AF) and SVD-related lacunar stroke. While the former caused approximately two-thirds of recurrent IS in our study, one-third was caused by SVD (selectively only in patients with nonlobar ICH). While DOACs are used to prevent cardioembolism, SVD-related IS was also less frequent in the DOAC group, leading to an overall strong reduction of ISs in patients with nonlobar ICH and AF seen in PRESTIGE-AF. Other than ICH location, no other particular neuroimaging patterns were related to the risk of IS in this study.

The main strengths of our work include a prospective RCT coupled with the centralized imaging analysis. Major limitations include the modest number of outcome events, leading to statistical uncertainties. We were thus not able to correct for co-factors or perform analyses according to treatment allocation (which would not be meaningfully possible because almost all recurrent ICH events occurred in the DOAC group and most ISs occurred in the no-anticoagulation group). We were neither able to correct for multiple comparisons, which may have resulted in chance findings. Longer follow-up would have allowed detection of more events, leading to improved statistical precision. Only 54% of patients had available MRI, which limited analyses of MRI-specific markers and the assessment of CAA. A certain inclusion bias in the PRESTIGE-AF toward smaller and nonlobar ICH needs to be noted, although underlying SVD seemed rather severe. The classification of recurrent stroke was based on local clinical and radiologic reports rather than centralized neuroimaging analysis.

In conclusion, our analysis identifies potential subpopulations at particular risk of DOAC initiation (patients with cSS, old macrohemorrhages, and lacunes) and one at potentially particular benefit (patients with nonlobar ICH). However, owing to the limited number of patients and outcome events, these results will be need to confirmed in larger, ideally individual patient-data meta-analyses, as are already envisioned in the COCROACH collaboration.

## Appendix Coinvestigators

**Table:**

Coinvestigators are listed at Neurology.org/N.

## Glossary

AF: atrial fibrillation
CAA: cerebral amyloid angiopathy
cSS: cortical superficial siderosis
DOAC: direct oral anticoagulant
HR: hazard ratio
ICH: intracerebral hemorrhage
IQR: interquartile range
IS: ischemic stroke
RCT: randomized controlled trial
SVD: small vessel disease
